# Sixteenth Annual Meeting of the Mississippi Valley Association of Dental Surgeons

**Published:** 1860-04

**Authors:** Geo. Watt


### Proceedings of Societies.

### SIXTEENTH ANNUAL MEETING OF THE MISS. VALLEY ASSOCIATION OF DENTAL SURGEONS.

MINUTES.

THE Association met according to adjournment, in the Ohio College of Dental Surgery, February 22, 1860. The President being absent, the Vice President, Dr. H. R. Smith, presided.

Members present: Drs. H. R. Smith, J. Taft, S. B. Smith, Joseph Richardson, James Taylor, A. G. Stipher, J. W. Baxter, C. N. Woodward, C. Bonsall, H. A. Smith, J. P. Ulrey, and G. Watt.

The minutes of the last meeting were read and approved. The Executive Committee being absent, on motion of Dr. Taft, the Chair was empowered to fill the same, which was done by the appointment of Drs. Taft, Taylor and Baxter, who immediately retired for consultation, and in a few minutes presented the following report:

REPORT OF THE EXECUTIVE COMMITTEE.

The Executive Committee would respectfully submit the following report on the order of business:

I. Reports of Committees.
II. Reports of Officers.
III. Reading of Essays and Papers.
IV. Election of Officers.
V. Miscellaneous business, including the following subjects for discussion:

1. Mechanical Dentistry,
Continuous Gum Work,
Vulcanite Base,
Cheoplasty,
Gold Work.

2. What is the best treatment when the pulp of a tooth is exposed, in order to secure its vitality?

3. Filling Teeth.

4. Irregularities of the teeth, their causes and modes of treatment.

5. Structure and nutrition of dental tissues.

6. Hemorrhage following extraction of the teeth, and best treatment. Respectfully submitted,

J. TAFT,

JAS. TAYLOR,

J. W. BAXTER, } Committee, *pro tem.*

The report was accepted and adopted, and the Committee discharged.

The Committee on Membership presented the names of Drs. Wm. H. Atkinson, of Cleveland, Ohio; J. F. Johnston, Indianapolis, Indiana; George Lupton, Shelbyville, Indiana; J. A. McClelland, Louisville, Ky.; Geo. F. Foote, T. F. Davenport, Cincinnati, Ohio, and Stoddard Driggs, Lexington, Ky., as candidates for membership, who, on being severally balloted for, were declared unanimously elected.

The Committee on Dental Progress had no report and was on motion, excused, as was also the Committee on Irregularity.

The Treasurer presented the following report, and requested that a committee be appointed to audit the same:

The Treasurer would report the following as the state of accounts at this time:

| | | |
|---|---|---|
| Balance in the Treasury, Feb. 22, 1859 | | $153 00 |
| Receipts from Initiation fees and Annual dues | | 74 00 |
| | | $227 00 |
| 1859. | | |
| Feb. 25, '59, To cash paid Dr. Watt for reporting as per order of Association | $15 00 | |
| To cash paid janitor for services as per order of Association | 8 00 | $023 00 |
| Balance | | 204 00 |

In addition, the Treasurer would report the following names of gentlemen elected to membership last year, but who had not yet paid their initiation fees, viz: Drs. J. B. Doughtry, of Monticello, Miss.; G. B. Lenoir, also of Monticello, Miss.; O. Wilson, of Aurora, Illinois, and B. B. Ward, Mobile, Alabama.

All of which is respectfully submitted,

*Feb.* 22, 1860, CHARLES BONSALL.

In compliance with the Treasurer's request, the Chair appointed Drs. Atkinson and Stipher, a committee to audit the report, who after due examination reported as follows:

The Auditing Committee, having examined the Treasurer's accounts, would report them as correct.

W. H. Atkinson, }

A. G. Stipher. } Committee.

On motion of Dr. Taft, the election of officers was made the order of the day for 11½, A. M., to-morrow.

Miscellaneous business being now in order the discussion of No. 1 was begun, and while it was still pending, on motion, adjourned till 2½, P. M.

FIRST DAY—AFTERNOON SESSION.

Met according to adjournment. The President and Vice President, both being absent, Dr. W. H. Atkinson was elected President, *pro. tem.* The discussion of No. 1 was resumed, the consideration of its subdivisions occupying the afternoon. The Vice President soon arrived and took the chair. Adjourned till 10, A. M., to-morrow.

SECOND DAY—MORNING SESSION.

Met according adjournment, the Vice President in the chair.

On motion of Dr. Watt, the resolution providing for a Standing Committee on Dental Progress was repealed. Proceeded to the discussion of Gold Work, the last subdivision of No. 1.

It being the order of the day, proceeded to the election of officers, which resulted as follows:

W. H. Atkinson, *President.*
J. F. Johnston, *Vice President.*
Joseph Richardson, *Corresponding Secretary.*
Geo. Watt, *Recording Secretary.*
Chas. Bonsall, *Treasurer.*
Geo. F. Foote, T. F. Davenport, Stoddard Driggs, *Executive Committee.*
J. Taft, J. W. Baxter, J. A. McClelland, *Committee on Membership.*

On motion of Dr. Watt, the resolution providing for a Standing Committee on Irregularities of the Teeth was repealed.

Dr. TAFT made, by consent, some remarks on the importance of microscopic researches, and after some general remarks by Dr. Atkinson and others, Dr. Johnston offered the following resolution, which, after due consideration, was adopted:

*Resolved*, That this association appropriate one hundred dollars for the purpose of aiding in the purchase of a microscope, to be used conjointly by this association and the Ohio Dental College, provided the faculty of said college contribute a like, or a greater amount of funds to this end and for that purpose.

Adjourned till 2½, P. M.

SECOND DAY—AFTERNOON SESSION.

Met according to adjournment, Vice President in the chair. Drs. H. A. Smith, Wm. H. Atkinson and Geo. F. Foote, were appointed to read essays at the next annual meeting. It was moved and carried, that a committee of two be appointed to carry out the spirit of the resolution relative to the purchase of a microscope. Drs. Atkinson and Taft were appointed said committee.

The Vice President appointed Dr. Baxter a committee to conduct the President elect to the chair, who immediately discharged the duties. On taking the chair President Atkinson made some highly appropriate and practical remarks, which were well received by the association.

On motion of Dr. Watt, it was resolved that a committee of two be appointed to report at the evening session, on the best practical mode of obtaining a standard treatise or essay on the subject of Irregularity of the teeth. The Chair appointed Drs. Watt and Taft said committee. A letter from Dr. John G. Hamill was received, and read by the Secretary, and, on motion, placed on file. The remainder of the afternoon was spent in the discussion of No. 2.

On motion, adjourned till 7½, P. M.

SECOND DAY—EVENING SESSION.

Met according to adjournment. The committee appointed to report relative to an essay on Irregularity reported as follows:

Your Committee, in view of the great importance of the subject under consideration, would respectfully recommend

1. That an award of fifty dollars be offered by the association for the best treatise or essay on Irregularity of the teeth.

2. That a committee of five be elected by ballot to examine the treatises submitted and make the award.

3. That documents competing for said award must be placed in the hands of the chairman of said committee, as early as January 1st, 1861.

4. That the author's name, in a sealed envelop, accompanying each document, which envelop must not be opened, unless the accompanying essay receive the award, and no essay may be shown to any person not a member of the committee, till after the association has accepted and adopted the report of said committee, when, of course, the successful treatise becomes the property of the association.

5. That rejected documents be disposed of according to accompanying directions.

6. That this offer and its conditions be published in all the dental journals of America.

7. That if the committee believe that none of the essays presented are worthy of the award, they have power to reject all of them, and so report to the association

Respectfully submitted.

GEO. WATT,

J. TAFT. } Committee.

The report was accepted and the committee discharged. A motion was made to adopt the report which was lost.

*Report of the Committee on Prize Essays.*—Your committee would most respectfully submit the following report on the Prize Essay, that about one year ago the third edition of the Prize Essay was issued. It consisted of one thousand copies bound in paper, five hundred copies bound in flexible cloth, two hundred in fine cloth binding and gilt; cost of the entire edition was $129 00. The entire value of the edition, at retail price, was $170 00. There is remaining on hands of the books to the amount of $16 00, leaving a net profit of $25 00 over and above the cost of the edition, together with the $16 00 worth of books on hands, making a net profit on the whole edition of $41 00.

Your committee would also suggest that another edition is already called for, and would suggest a revision of the work, by the author.

J. TAFT.

J. RICHARDSON. } Committee.

The report was accepted and the committee continued with the same powers.

The remainder of the session was occupied with the discussion of No. 3.

Adjourned till 9, A. M., to-morrow.

THIRD DAY—MORNING SESSION.

Met according to adjournment. President in the chair. Proceeded to the discussion of No. 4. No. 5 was continued till the next annual meeting, with a request that the Executive Committee give it a prominent place in next year's programme. No. 6 was discussed, after which, by request, the President read a paper on the "Relation of the Physician and Dentist." A cordial vote of thanks was given for the paper; but, as it was written expressly for a medical journal, a copy for publication was not requested.

On motion of Dr. Watt, Drs. J. Taylor and H. R. Smith, were added to the list of essayists, the reason for the motion being stated, that the association had lost standing by a neglect of this important department of its proceedings.

The Minutes were read and approved. On motion adjourned to meet in the Dental College, Wednesday, February 20th, 1861, at 10 o'clock, A. M.

GEO. WATT, *Secy.*

---

